# Efficacy and safety of laparoscopic bariatric surgery in patients of 70 years and older: A systematic review and meta‐analysis

**DOI:** 10.1111/obr.13867

**Published:** 2024-11-17

**Authors:** Jan Kapała, Tomasz Maroszczuk, Natalia Dowgiałło‐Gornowicz

**Affiliations:** ^1^ Department of General, Minimally Invasive and Elderly Surgery, Collegium Medicum University of Warmia and Mazury Olsztyn Poland

**Keywords:** 70 years old, bariatrics, elderly, systematic review

## Abstract

**Introduction:**

Aging population and growing obesity prevalence are two major public health issues. Bariatric surgery has been shown to be both safe and effective, but its role in the treatment of the elderly remains controversial.

**Objectives:**

To evaluate the efficacy and safety of laparoscopic bariatric surgery in patients over 70 years of age.

**Methods:**

A systematic review and assessment of the literature was performed in November–December 2023. Inclusion criteria gathered studies of elderly (age ≥70 years old) who underwent laparoscopic bariatric surgery. The data extraction focused on weight loss, obesity‐related diseases, and complications.

**Results:**

Fourteen retrospective studies were included, involving 3923 septuagenarians (female, 69.70%). One year after the surgery, the mean excess weight loss was 54.66%. At last follow‐up, the improvement in obesity‐related diseases was regarded as 50% diagnosed with diabetes, 36% with hypertension, 50% with reflux, 36% with sleep apnea, and 25% with hyperlipidemia. The overall postoperative major morbidity and mortality were about 2% and 1%, respectively.

**Conclusions:**

This systematic review suggests that laparoscopic bariatric surgery is an effective and safe treatment for patients over 70 years of age.

**Abbreviations**
ACS‐NSQIP: American College of Surgeons National Surgical Quality Improvement Program
AF: atrial fibrillation
BMI: body mass index
CCS: case–control study
CI: confidence interval
CI l.: lower bound of confidence interval
CI u.: upper bound of confidence interval
CMS: case‐matched study
D: mean difference
DS: duodenal switch
EWL: excess weight loss
GBP: gastric bypass
GERD: gastroesophageal reflux disease
HLD: hyperlipidemia
HT: hypertension
LAGB: laparoscopic adjustable gastric banding
MINORS: Methodological Index for Non‐Randomized Studies
N: number of participants
N/A: not available
OAGB: one anastomosis gastric bypass
OR: odds ratio
OSA: obstructive sleep apnea
PRISMA: Preferred Reporting Items for Systematic Reviews and Meta‐Analyses
RS: retrospective study
RYGB: Roux‐en‐Y gastric bypass
SG: sleeve gastrectomy
T2D: type 2 diabetes
TWL: total weight loss

## 1 INTRODUCTION

Aging population and growing obesity prevalence are two major public health issues. In the United States and Europe, the obesity rate is projected to peak between 2030 and 2060. Age‐specific obesity rate follows an inverted U‐shape, culminating between the ages of 60 and 69.^1^ In addition, a considerable demographic shift will occur by 2050. By then, in nearly 30 countries, the proportion of individuals aged 65 and above will exceed 28% of the total population.^2^

Presented trends lead to an increase in the number of elderly patients suffering from obesity and obesity‐related diseases such as type 2 diabetes (T2D), hypertension (HT), obstructive sleep apnea (OSA), gastroesophageal reflux disease (GERD), or hyperlipidemia (HLD).^3^ The consensus remains that weight loss in the elderly includes a number of reported benefits.^4^ Bariatric surgery has been shown to be safe and effective for long‐term weight loss maintenance and the control of obesity‐related disease. While reviews and meta‐analyses of bariatric surgery in the elderly have already been conducted, they typically analyze groups of patients under 60 or 65 years of age.^5^, ^6^ Due to the increasing number of studies comparing bariatric surgery effectiveness and safety in patients under and over 70 years of age, we believe there is a need for a review and synthesis of those studies.

The purpose of this systematic review was to highlight the positive and negative effects of laparoscopic bariatric surgery among patients of 70 years and older and to assess the effectiveness and safety of this procedure.

## 2 MATERIALS AND METHODS

### 2.1 Data sources

The literature review and assessment were performed systematically in November–December 2023. Four databases (PubMed, Cochrane Library, Google Scholar, and ClinicalTrials) were searched using the following algorithm: [(elderly” OR “70 years old”) AND (“bariatric surgery” OR “sleeve gastrectomy” OR “gastric bypass” OR “gastric band”) AND “laparoscopy”]. According to the PICO framework, this review included: Patients: “people with obesity over 70 years of age”; Intervention: “laparoscopic bariatric surgery”; Comparison: “people with obesity people under 70 years of age”; and Outcomes: “weight loss,” obesity‐related diseases improvement, and complications incidence.” We chose a cut‐off of 70 years old, as we observed that there is a lack of literature systematically analyzing the effectiveness and safety of bariatric surgery in this age cohort.

### 2.2 Inclusion and exclusion criteria

All human clinical trials and studies reporting outcomes of elderly (≥70 years old) undergoing laparoscopic bariatric surgery were identified. We included articles published in English until December 2023 without any restrictions on participants' demographic, quantitative, gender, race, or country of origin. Only full texts were accessed for review. We included only trials in which all procedures involving human participants were conducted in accordance with the ethical standards of the institutional or national research committee and with the 1964 Helsinki declaration and its later amendments or comparable ethical standards.

### 2.3 Outcome definitions

Outcomes in weight loss were evaluated with body mass index (BMI) and excess weight loss (EWL). “Baseline” refers to the day of surgery. Bariatric success was defined as ≥ 50% EWL.^7^ The obesity‐related diseases were assessed as persisting (the same medications as before surgery) or improving (reduction in medications). The early mortality/complications were defined as deaths or events occurring in the 30 days after the surgery or during the hospitalization. Similarly, the late mortality/complications were defined as death or events occurring over 30 days after the surgery. Major morbidity was defined as the presence of one or more of the following postoperative events: unplanned intubation or failure to wean from ventilator, pulmonary embolism, progressive renal insufficiency, acute renal failure, cerebrovascular accident, myocardial infarction, or sepsis.

### 2.4 Data reviewing and extraction

This systematic review was performed in accordance with Preferred Reporting Items for Systematic Reviews and Meta‐Analyses (PRISMA) guidelines.^8^ Two authors (JK, TM) independently reviewed databases, read titles, abstracts, and full‐text articles selected to extract study data. Any disagreements were resolved by third‐author consensus (NDG). Data collection was performed with emphasis on the author of the study, year of publication, study design, study population, and type of bariatric surgery. It also included follow‐up, gender, age, baseline BMI, %EWL 6 and 12 months after surgery, pre‐ and post‐operative occurrence of obesity‐related diseases (T2D, HT, GERD, OSA, and HLD), operative time, length of hospital stay, specific early and late postoperative complications, and mortality.

The main primary interest was to collect results from articles concerning bariatrics in the elderly (≥70 years old) and to summarize its clinical efficacy and adverse event profile with younger peers. However, because of the lack of placebo groups or insufficient data in this area and the high heterogeneity of outcomes measurement in the included studies, the basic form of data processing—meta‐analysis—was highly limited and partially replaced by narrative synthesis. Results were grouped into three categories: weight loss, obesity‐related diseases' improvement, and complications. If some results were missing, the authors tried to calculate them manually whenever possible.

The quality of the included studies and the potential risk of bias were assessed using the Methodological Index for Non‐Randomized Studies (MINORS).^9^ A satisfactory MINORS score exceeded 8 points for a non‐comparative study and 12 points for a comparative study.

### 2.5 Statistical analysis

All statistical analysis was carried out using the “meta‐analysis and meta‐regression” tool in Statistica (data analysis software system), version 13, http://statistica.io (accessed March 25, 2024), TIBCO Software Inc., Krakow, Poland (2017). According to the type of the available data (“2 x 2 tables” or “means – independent groups”), odds ratio (OR) or mean difference (D) along with their two‐sided 95% confidence intervals (CIs) were used as the measure of the effect. Heterogeneity analysis was performed using the Cochrane test (Q, *I* ^2^, and *p*‐value) based on *X* ^2^ and tau‐squared test (*τ* ^2^). Publication bias and symmetry of funnel plots were analyzed using Begg and Mazumdar's rank correlation test and Egger's regression. In all statistical tests, *p* values below 0.05 were regarded as significant, and those between 0.05 and 0.1 were considered almost significant. To demonstrate the data from multiple studies observing the same effect, the forest plot blobbogram was used.

## 3 RESULTS

### 3.1 Study selection and characteristics

**FIGURE 1:**
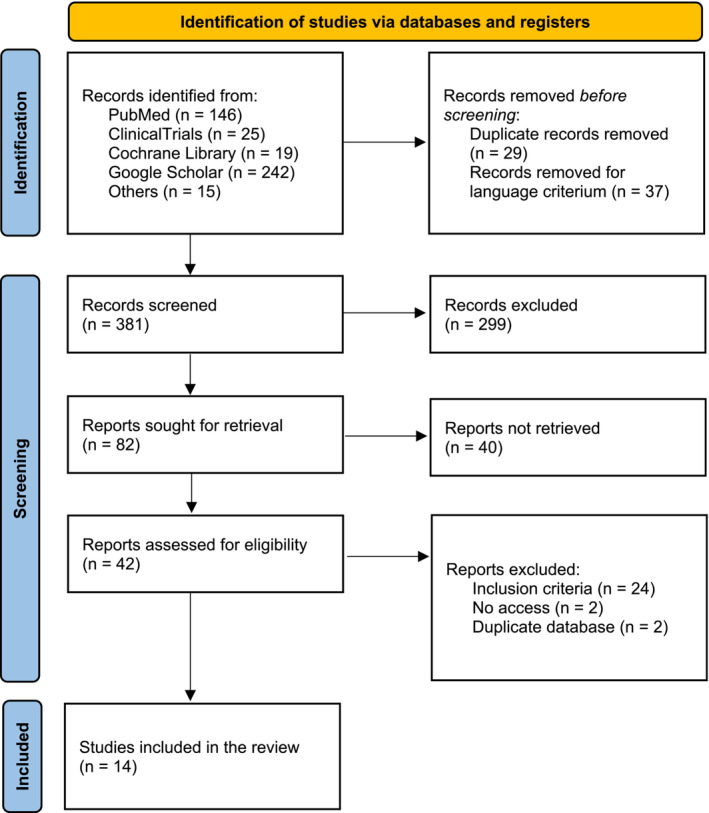
Preferred Reporting Items for Systematic Reviews and Meta‐Analyses (PRISMA) flow diagram of literature search and selection process.^8^

During the literature review, authors identified 447 studies, of which 42 were assessed for full‐text eligibility. A total of 405 studies were rejected earlier: 29 duplicates, 37 removed for language criterion, 299 excluded because of the study design, and absence of the main primary outcome. Of the 42 reports assessed for eligibility, 24 did not meet inclusion criteria, two had access issues, and two had duplicate databases (Figure 1).

Finally, 14 retrospective studies were included^10^, ^11^, ^12^, ^13^, ^14^, ^15^, ^16^, ^17^, ^18^, ^19^, ^20^, ^21^, ^22^, ^23^: five of them were non‐comparative^10^, ^13^, ^15^, ^17^, ^20^ and nine were comparative studies.^11^, ^12^, ^14^, ^16^, ^18^, ^19^, ^21^, ^22^, ^23^ Two of them were case‐matched studies.^11^, ^12^ The results of the review are presented in Table 1, providing a summary of included studies and their outcomes. Of the 14 studies, which included a total of 3923 patients over the age of 70 who underwent laparoscopic bariatric surgery: 1307 (33%) had sleeve gastrectomy (SG), 1057 (27%) had Roux‐en‐Y gastric bypass (RYGB), 105 (3%) had laparoscopic adjustable gastric banding (LAGB), 15 (<1%) had duodenal switch (DS), 2 (<1%) had gastric plication, and for 1437 (37%) the type of surgery was unknown. Based on known patients' ages, the mean age of these patients was 71.72 years. There were 2655 female (69.70%) and 1154 male (30.30%). The mean follow‐up was 23.07 months and ranged from one to 219 months.

**TABLE 1:** Summary of included studies: efficacy and safety of laparoscopic bariatric surgery in patients >70.

References	Study design	*N*	Gender (female/male)	Age [years]	Type of surgery	Follow‐up [months]	MINORS score
Zaveri 2016^10^	RS	53	35/18	Mean 72.7 ± 2.4 (range, 70–81.4)	LAGB, RYGB, SADS	18	12/16
Parmar 2017^11^	CMS	10	7/3	Mean 71 (range, 70–77)	RYGB, SG, OAGB	Mean 22 (range, 6–24)	16/24
Hansel 2023^12^	CMS	1307	1004/303	Mean 70.6 ± 1.9	N/A	Mean 48 ± 24	18/24
Hammond 2020^13^	RS	23	N/A	Mean 72 (range, 70–80)	RYGB	Median 17 (range, 1–219)	11/16
Pechman 2019^14^	RS	1495	991/504	Mean 72.5 ± 3.0 (range, >70)	SG, RYGB	1	17/24
Nor Hanipah 2018^15^	RS	19	8/11	Median 76 (range, 75–81)	SG, LAGB, RYGB, gastric plication	Median 48 (range, 12–120)	9/16
Goldenberg 2022^16^	CCS	25	23/2	Mean 71.9 (range, 70–78)	SG	12	16/24
Loy 2014^17^	RS	55	33/22	Mean 72.4 ± 2.5	LAGB	96	10/16
Athanasiadis 2021^18^	RS	29	23/6	Mean 72 ± 1.7	RYGB, SG	48	15/24
Al‐Kurd 2018^19^	RS	30	15/15	Mean 71.9 ± 2.3	SG	12	15/24
Ramirez 2012^20^	RS	42	22/20	Mean 73.5 (range, 71–80)	LAGB, SG, RYGB	12	9/16
Belluzzi 2023^21^	RS	103	71/32	Mean 72.1 ± 2.5	SG, RYGB	60	17/24
Smith 2019^22^	RS	641	423/218	Mean 72.4 ± 2.0	SG, RYGB	12	14/24
Susmallian 2019^23^	RS	91	N/A	Range >70	SG, GBP, RYGB, OAGB, LAGB, DS	Range, 9–45	14/24

Abbreviations: CCS, case–control study; CMS, case‐matched study; DS, duodenal switch; GBP, gastric bypass; LAGB, laparoscopic adjustable gastric banding; MINORS, Methodological Index for Non‐Randomized Studies; *N*, number of participants; N/A, not available; OAGB, one anastomosis gastric bypass; RS, retrospective study; RYGB, Roux‐en‐Y gastric bypass; SG, sleeve gastrectomy.

According to the MINORS scale, the average value for non‐comparative studies exceeded 10/16 and for comparative studies 15/24 (Table 1). Therefore, all studies were of high quality and minimal risk of bias.

### 3.2 Weight loss

The mean baseline BMI was 42.82 kg/m^2^ (12 studies) and ranged from 35.9 to 63.7 kg/m^2^. The mean %EWL at 6 months was 39.72% (5 studies), with a range of 39.3–102.2%, and at 1 year it was 54.66% (10 studies), ranging 21%–109.6%. This suggests that most patients significantly reduced their weight within 1 year. The effectiveness of treatment could not be assessed because of the absence of patient‐specific data. However, it can be presumed that the majority of patients had bariatric success at ≥50%EWL, given that the mean first‐year %EWL was 54.66% (Table 2).^7^

**TABLE 2:** Weight loss after bariatric surgery in patients >70 years old.

References	*N*	BMI (baseline) [kg/m^2^]	%EWL (6 months after surgery)	%EWL (1 year after surgery)
Zaveri 2016^10^	53	Mean 43.35 ± 5.57	Mean 41.31	Mean 51.05
Parmar 2017^11^	10	Mean 48.4 (range, 39.3–54.6)	Mean 67.4 (range, 39.3–102.2)	Mean 74.0 (range, 40.5–109.6)
Hansel 2023^12^	1307	Range, >30	N/A	N/A
Hammond 2020^13^	23	Mean 43.3 (range, 37.3–56.0)	N/A	Mean 60 (range, 21–105)
Pechman 2019^14^	1495	Mean 41.3 ± 8.5	N/A	N/A
Nor Hanipah 2018^15^	19	Median 41.4 (range, 35.8–57.5)	N/A	Median 47.1
Goldenberg 2022^16^	25	Mean 43.4 (range, 37.6–63.7)	N/A	Mean 65.1 (range, 49.3–91.9)
Loy 2014^17^	55	Mean 45	Mean 27 ± 6.7	Mean 36 ± 12.7
Athanasiadis 2021^18^	29	Mean 41.1 ± 6.8	N/A	N/A
Al‐Kurd 2018^19^	30	Mean 42.5 ± 5.4	N/A	N/A
Ramirez 2012^20^	42	Mean 46.8 ± 9.3	Mean 42.7	Mean 47.7
Belluzzi 2023^21^	103	Mean 47.7 ± 6.8	Mean 41.79 ± 12.86	Mean 51.1 ± 17.51
Smith 2019^22^	641	Mean 45.1 ± 6.8	N/A	Mean 59.3
Susmallian 2019^23^	91	N/A	N/A	Mean 37.8

Abbreviations: BMI, body mass index; *N*, number of participants; N/A, not available; %EWL, percentage of excessive weight loss.

**FIGURE 2:**
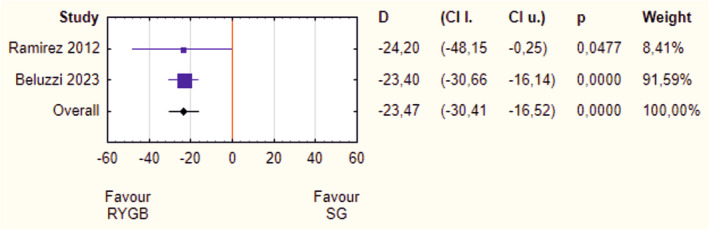
Forrest plot blobbogram—percentage of excess weight loss (%EWL) after 1 year: sleeve gastrectomy (SG) versus Roux‐en‐Y gastric bypass (RYGB). CI l., lower bound of confidence interval; CI u., upper bound of confidence interval; D, mean difference.

Due to the lack of data (e.g., means, standard deviations) and the high heterogeneity of outcome measures in the studies included (e.g., different weight loss rates), the meta‐analysis on weight loss was highly limited. The only analysis possible to perform was the comparison of %EWL 1 year after surgery in septuagenarians between two surgeries: SG and RYGB. Our results favor RYGB (D = −23.47, 95% CI = −30.41 to −16.52, *p* < 0.001) (Figure 2). There was no heterogeneity (*I* ^2^ ~0%) or asymmetry (*p* > 0.05).

### 3.3 Obesity‐related diseases improvement

Before the surgery, some patients were diagnosed with obesity‐related diseases: 1656 (43%; 13 studies) had T2D, 2923 (76%; 13 studies) had HT, 25 (30%; 2 studies) had GERD, 1269 (56%; 9 studies) had OSA, and 752 (78%; 9 studies) had HLD. After the bariatric procedure, at the last follow‐up, a considerable number of septuagenarians experienced improvement, expressed by discontinuation of pharmacological treatments or reduction of doses. This improvement regards 81 (50%; 9 studies) diagnosed with T2D, 102 (36%; 9 studies) with HT, 10 (50%; 1 study) with GERD, 60 (36%; 4 studies) with OSA, and 52 (25%; 7 studies) with HLD (Table 3).

**TABLE 3:** Obesity‐related diseases improvement after bariatric surgery in patients >70 years old.

References	*N*	T2D before/after (%)	HT before/after (%)	GERD before/after (%)	OSA before/after (%)	HLD before/after (%)
Zaveri 2016^10^	53	25/5 (20%)	42/11 (26%)	20/10 (50%)	31/15 (48%)	N/A
Parmar 2017^11^	10	5/0 (0%)	8/0 (0%)	N/A	N/A	N/A
Hansel 2023^12^	1307	396/N/A	795/N/A	N/A	676/N/A	N/A
Hammond 2020^13^	23	17/5 (29%)	22/16 (73%)	N/A	N/A	17/11 (65%)
Pechman 2019^14^	1495	718/N/A	1251/N/A	N/A	N/A	N/A
Nor Hanipah 2018^15^	19	8/2 (25%)	8/1 (13%)	N/A	11/N/A	7/7 (0%)
Goldenberg 2022^16^	25	14/2 (14%)	20/4 (20%)	N/A	N/A	21/11 (52%)
Loy 2014^17^	55	23/15 (65%)	49/36 (73%)	N/A	31/20 (65%)	40/29 (73%)
Athanasiadis 2021^18^	29	15/12 (80%)	27/24 (89%)	N/A	18/13 (72%)	25/20 (80%)
Al‐Kurd 2018^19^	30	16/12 (75%)	19/9 (47%)	5/N/A	12/N/A	16/9 (56%)
Ramirez 2012^20^	42	16/N/A	28/N/A	N/A	11/N/A	24/N/A
Belluzzi 2023^21^	103	38/27 (71%)	90/82 (91%)	N/A	88/60 (68%)	82/69 (84%)
Smith 2019^22^	641	365/N/A	564/N/A	N/A	391/N/A	520/N/A
Susmallian 2019^23^	91	N/A	N/A	N/A	N/A	N/A

Abbreviations: GERD, gastroesophageal reflux disease; HLD, hyperlipidemia; HT, hypertension; *N*, number of participants; N/A, not available; OSA, obstructive sleep apnea; T2D, type 2 diabetes.

**FIGURE 3:**
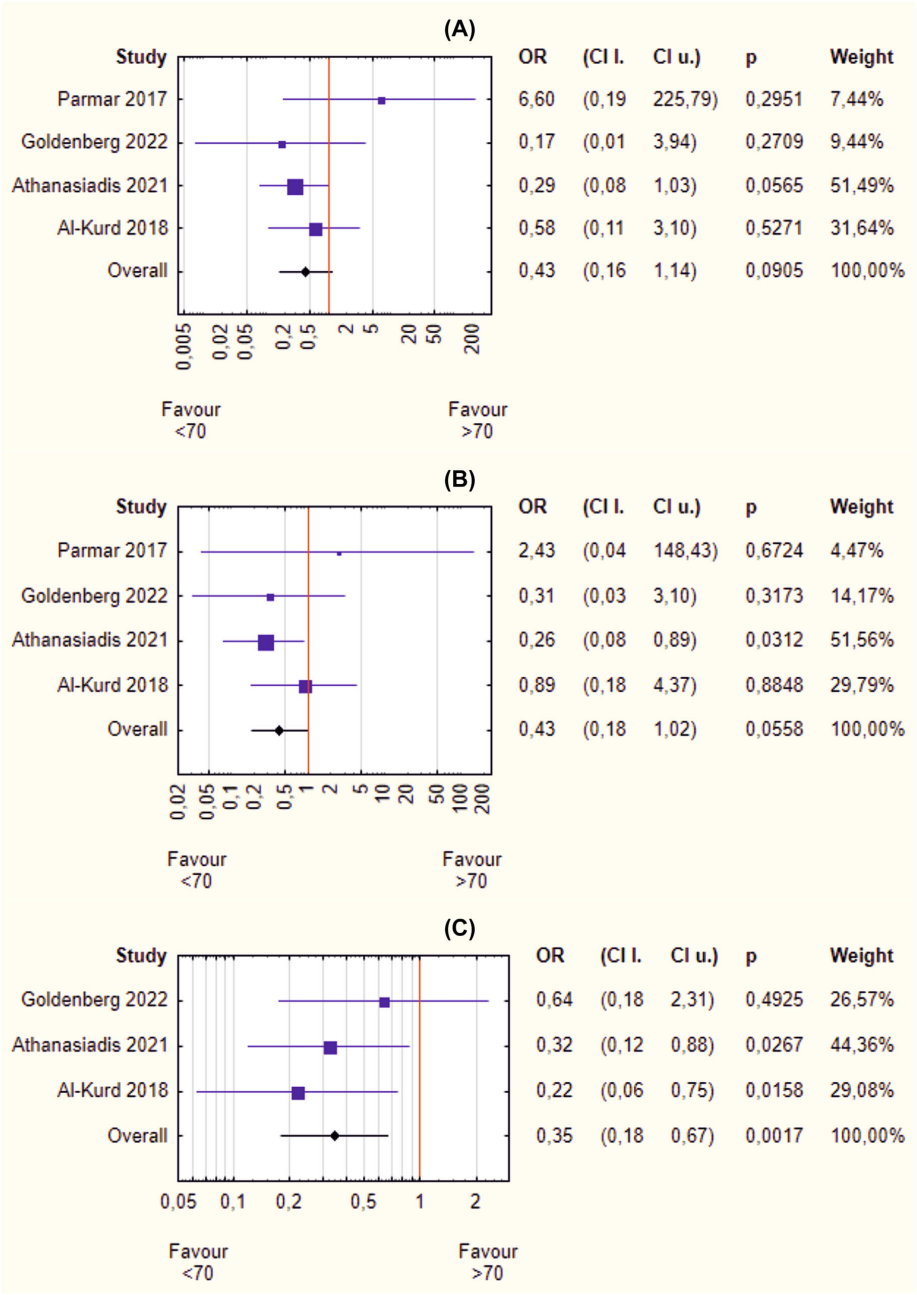
(A–C) Forrest plot blobbogram—obesity‐related diseases improvement after bariatric surgery in patients under and over 70 years of age: type 2 diabetes (A), hypertension (B), hyperlipidemia (C). CI l., lower bound of confidence interval; CI u., upper bound of confidence interval; OR, odds ratio.

Based on available data on the improvement of obesity‐related diseases, it was possible to conduct a meta‐analysis in three of five diseases: T2D, HT, and HLD. For patients with diabetes, younger peers had slightly better results, but these findings were only nearly significant (OR = 0.43, 95% CI 0.16–1.14, *p* = 0.0905) (Figure 3). Similarly, HT treatment outcomes slightly favor patients under 70 years of age, yielding a nearly significant difference (OR = 0.43, 95% CI 0.18–1.02, *p* = 0.056) (Figure 3B). Only resolution of HLD occurred significantly more often in younger patients (OR = 0.35, 95% CI 0.18–0.67, *p* = 0.002) (Figure 3C). Neither heterogeneity (*I* ^2^ ~0%) nor asymmetry (*p* > 0.05) was observed.

### 3.4 Postoperative complications

The mean operative time of a performed bariatric procedure was equal to 107.36 min (5 studies), and the mean length of hospital stay was 2.81 days (10 studies).

Specific early complications included: atrial fibrillation (AF) (*n* = 3; 16%; 1 study), emergency room visit (*n* = 59; 9%; 2 studies), band removal (*n* = 3; 7%; 1 study), readmission (*n* = 34; 5%; 2 studies), accidental colotomy (*n* = 2; 5%; 1 study), infection (*n* = 104; 4%; 7 studies), failure‐to‐thrive requiring temporary enteral nutrition (*n* = 1; 4%; 1 study), postoperative nausea and vomiting (*n* = 1; 4%; 1 study), reoperation (*n* = 61; 3%; 3 studies), transfusion required intraoperatively or postoperatively (*n* = 40; 3%; 1 study), bleeding (*n* = 21; 3%; 3 studies), cardiac arrhythmia (*n* = 4; 2%; 3 studies), stricture (*n* = 1; 2%; 1 study), acute cholecystitis and subhepatic abscess (*n* = 1; 2%; 1 study), gastro‐cutaneous fistula (*n* = 1; 2%; 1 study), reflux (*n* = 1; 2%; 1 study), failure to wean from ventilator (*n* = 14; 1%; 1 study), thrombotic events (*n* = 13; 1%; 3 studies), leak or perforation (*n* = 11; 1%; 5 studies), septic shock (*n* = 9; 1%; 1 study), renal insufficiency (*n* = 12; 1%; 1 study), obstruction (*n* = 5; 1%; 1 study), stroke/cerebrovascular accident (*n* = 3; <1%; 1 study), myocardial infarction (*n* = 7; <1%; 2 studies). Early mortality rate was low—<1% (16 patients; 12 studies).

Specific late complications included: ulcer‐related complications (*n* = 9; 31%; 1 study), reflux (*n* = 9; 12%; 2 studies), poor oral intake (*n* = 2; 11%; 1 study), late reoperation (*n* = 1; 10%; 1 study), nausea and vomiting (*n* = 5; 9%; 1 study), weight regain (*n* = 4; 8%; 1 study), hypovitaminosis (*n* = 2; 8%; 1 study), diarrhea (*n* = 3; 6%; 1 study), recurrent AF (*n* = 1; 5%; 1 study), stricture (*n* = 2; 4%; 1 study), pancreatic cancer (*n* = 1; 4%; 1 study), epigastralgia (*n* = 1; 4%; 1 study), band slip (*n* = 3; 3%; 2 studies), internal hernia (*n* = 1; 3%; 1 study), gastrointestinal bleeding (*n* = 1; 3%; 1 study), erosion of lap band port (*n* = 1; 2%; 1 study), band removal (*n* = 1; 2%; 1 study), port site hernia (*n* = 1; 2%; 1 study), megaesophagus (*n* = 1; 2%; 1 study). Late mortality rate was about 4% (13 patients; 9 studies). The synthesized overall major morbidity and mortality were approximately 2% (39 patients; 10 studies) and 1% (29 patients; 12 studies), respectively.

**FIGURE 4:**
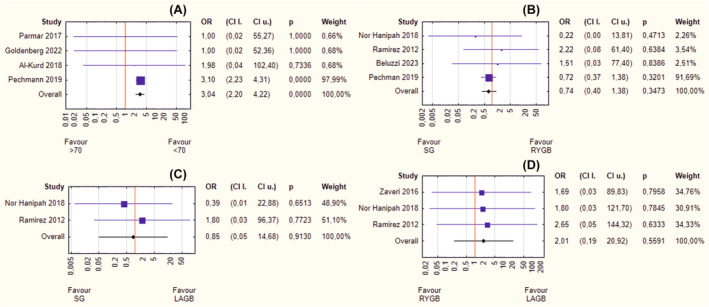
(A–D) Forrest plot blobbogram—postoperative major morbidity rate: septuagenarians versus younger peers (A), sleeve gastrectomy (SG) versus Roux‐en‐Y gastric bypass (RYGB) (B), SG versus laparoscopic adjustable gastric banding (LAGB) (C), RYGB versus LAGB (D). CI l., lower bound of confidence interval; CI u., upper bound of confidence interval; OR, odds ratio.

The meta‐analysis of the major morbidity rate showed significantly better results in younger peers (OR = 3.04, 95% CI 2.20–4.22, *p* < 0.001) (Figure 4). Any of the analyzed bariatric surgeries: SG versus RYGB (OR = 0.74, 95% CI 0.40–1.38, *p* = 0.347) (Figure 4B), SG versus LAGB (OR = 0.85, 95% CI 0.05–14.68, *p* = 0.913) (Figure 4C), and RYGB versus LAGB (OR = 2.01, 95% CI 0.19–20.92, *p* = 0.559) (Figure 4D) did not fare any better in terms of occurrence of serious complications. There was no heterogeneity (*I* ^2^ ~0%) or asymmetry (*p* > 0.05).

**FIGURE 5:**
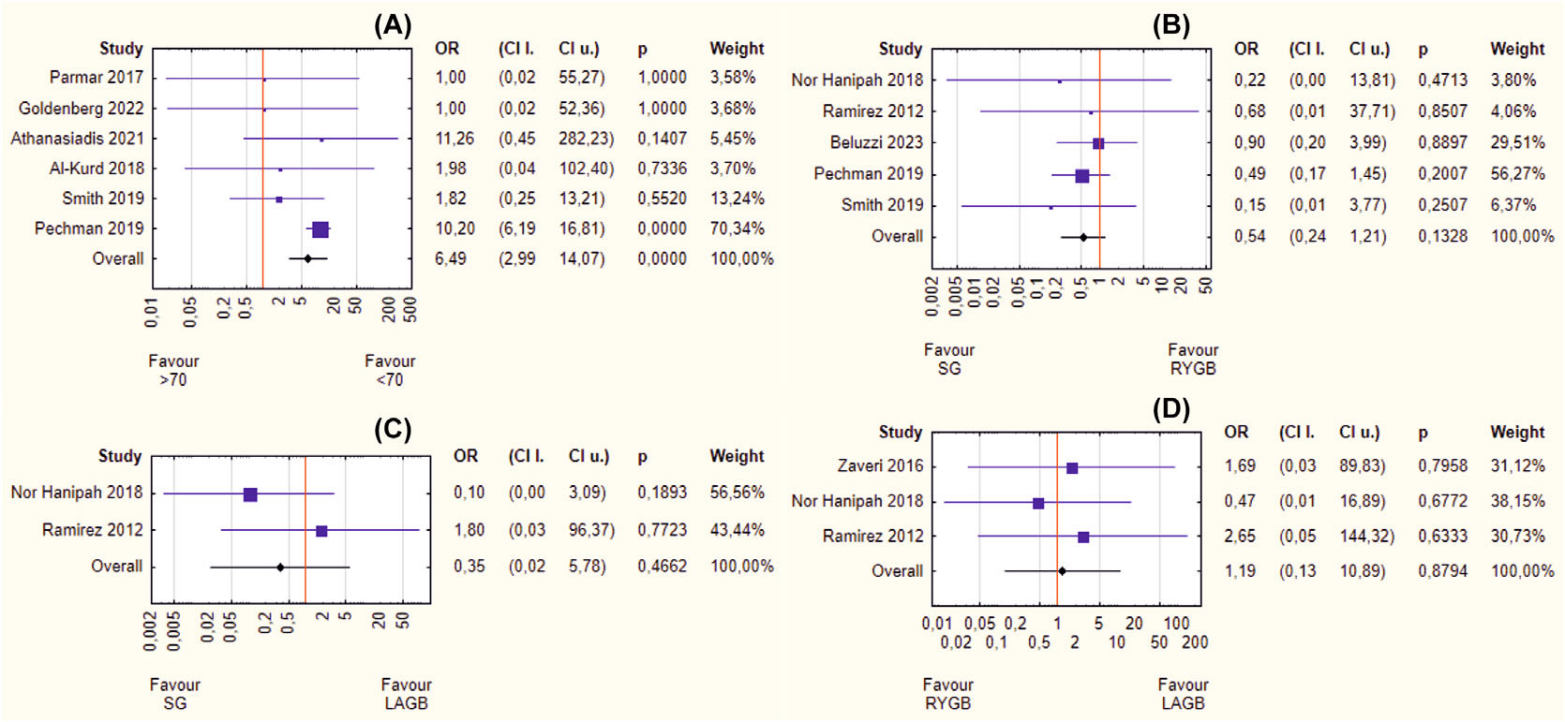
(A–D) Forrest plot blobbogram—postoperative mortality rate: septuagenarians versus younger peers (A), sleeve gastrectomy (SG) versus Roux‐en‐Y gastric bypass (RYGB) (B), SG versus laparoscopic adjustable gastric banding (LAGB) (C), RYGB vs. LAGB (D). CI l., lower bound of confidence interval; CI u., upper bound of confidence interval; OR, odds ratio.

Similarly, a meta‐analysis showed that mortality rate was significantly higher in patients over 70 years of age (OR = 6.49, 95% CI 2.99–14.07, *p* < 0.001) (Figure 5). No differences were found between individual surgical methods: SG versus RYGB (OR = 0.54, 95% CI 0.24–1.21, *p* = 0.133) (Figure 5B), SG versus LAGB (OR = 0.35, 95% CI 0.02–5.78, *p* = 0.466) (Figure 5C), and RYGB versus LAGB (OR = 1.19, 95% CI 0.13–10.89, *p* = 0.879) (Figure 5D). There was no heterogeneity (*I* ^2^ ~0%). The asymmetry (*p* < 0.05) was observed in the single analysis comparing mortality rates between patients younger and older than 70 years.

## 4 DISCUSSION

This systematic review was conducted to highlight the effects of bariatric surgery among patients over 70 years of age and evaluate its efficacy and safety in this population. Our study compares the results of 3923 patients aged 70 years or older who have undergone laparoscopic bariatric surgery procedures. To the best of our knowledge, this is the largest review made on this subject. Our findings reveal promising outcomes in terms of weight reduction, with a mean %EWL of 54.66%. The improvement in obesity‐related diseases regarded 51% diagnosed with diabetes, 34% with hypertension, 50% with esophageal reflux, 36% with sleep apnea, and 26% with hyperlipidemia. Septuagenarians have a higher incidence of major complications than younger patients, with infection, emergency room visit, readmission, and reoperation being the most widely reported among the studies. The overall postoperative mortality was about 1%.

Until recently, weight loss in elderly individuals with obesity caused concerns due to potential muscle loss and incline to frailty syndrome. Intentional weight loss, even when excess fat mass is targeted, may involve increased muscle loss, which has been found in older individuals to be negatively correlated with functional ability to live independently.^24^ Furthermore, some studies suggest weight loss in older adults may increase all‐cause mortality.^25^ In contrary to those studies, growing awareness of the health and functional consequences associated with extended obesity duration are coupled with evidence implying the safety of weight loss in this age group.^4^, ^26^ Lately a more proactive approach to weight loss was investigated, with a number of studies regarding bariatric surgery in elderly patients.^5^, ^6^, ^27^, ^28^ Furthermore, a recent study conducted on septuagenarians by Hansel et al showed that bariatric surgery, in addition to improving quality of life and aiding in the remission of obesity‐related diseases, can reduce morality as compared with an group with obesity not treated with bariatric surgery.^12^ The above‐mentioned studies have demonstrated the efficacy and safety of bariatric surgery as a means of weight loss for this demographic subgroup.

The mean percentage of EWL observed at the one‐year mark was 54.66%. Despite the lack of patient‐specific information, it is reasonable to deduce that a substantial portion of patients achieved bariatric success, defined as attaining at least a 50% reduction in excess weight, given the observed mean %EWL of 54.66% within the first year. Reported %EWL differed vastly in analyzed studies from %EWL equal to 36 ± 12.7 in a study by Loy et al. to %EWL equal to mean 74.0 (range, 40.5–109.6) in a study by Parmar et al.^11^, ^17^ It is vital to note that Parmar et al.'s study certainty is limited by the small number of participants.

The majority of analyzed studies present a relatively short follow‐up. The trajectory of weight loss after bariatric surgery assumes substantial significance in the context of long‐term bariatric success. Weight change dynamics between the first and second year after surgery is proven to be fundamental for maintaining lowered weight in the long term.^29^, ^30^, ^31^ Although studies that present follow‐ups regarding weight loss at least 2 years long are limited and the number of patients included in follow‐up falls significantly after the first year, they give valuable insight for possible long‐term effects of bariatric surgery in this age group.^11^, ^15^, ^18^, ^19^, ^21^

A study conducted by Athanasiadis et al. investigated the outcomes of bariatric surgery, specifically 4 years post‐operation among 29 septuagenarians.^18^ The findings indicated promising results, with reaching approximately 46%EWL. Similarly, Belluzzi et al. conducted a study on 109 patients, with follow‐up for 40 patients reaching 5 years after the operation. In this study, the %EWL of said 40 patients was reported as 44.3% ± 59.6% for SG patients and 55.3% ± 24.9% for RYGB patients.^21^ Additionally, Loy et al. conducted a study involving 15 patients after LAGB with 4‐year‐long follow‐ups, reporting a %EWL of 48% ± 22.6%.^17^ Data regarding long‐term weight loss effects among septuagenarians is limited, yet preliminary findings suggest promising outcomes.

The second major benefit of bariatric surgery is obesity‐related diseases remission. After the bariatric procedure, at the last follow‐up, a considerable number of septuagenarians experienced remission, expressed by discontinuation of pharmacological treatments or reduction of doses. The data gathered across the studies present improvement in obesity‐related diseases., Among all participants diagnosed with the stated disease, improvements were noted in: 51% diagnosed with T2D, 34% with HT, 50% with GERD, 36% with OSA, and 26% with HLD. Remission of diseases was observed across all the studies analyzing them, and as demonstrated by Hansel et al., it was likely the single most significant factor that decreased mortality when compared with individuals with obesity who had not undergone surgery. Our results show that the efficacy of obesity‐related diseases remission declines with age and slightly favors younger control groups.

One of the most crucial factors regarding acceptance and wide usage of certain operational treatments is how the potential benefits of said operations outweigh the potential risks. As demonstrated before, bariatric surgery offers septuagenarians the chance to achieve satisfactory obesity‐related diseases remission and weight loss. The question arises regarding the risks of bariatric surgery and how these risks compare with those associated with other surgical interventions conducted on elderly individuals. Pechman et al. and Smith et al. conducted major studies on this subject. Pechman et al. conducted the largest study on this subject with 1498 patients aged over 70 who underwent non‐revisional bariatric surgery, including 751 (50.1%) SG and 747 (49.9%) RYGB.^14^ While the study's nationwide database (American College of Surgeons National Surgical Quality Improvement Program [ACS‐NSQIP]) was not intended for bariatric parameter assessment, it presents great insight into complications and morbidity after a bariatric operation. Significant disparities between septuagenarians and a control cohort aged under 70 years old were observed in various parameters, including the duration from operation room admission to discharge (2.9 ± 4.8 days vs. 2.2 ± 2.4 days; *p* < 0.001), mortality rates (1% vs. 0.1%; *p* < 0.001), and morbidity rates (8% vs. 4.9%; *p* < 0.001). Furthermore, Pechman's investigation indicates heightened rates of specific adverse events, such as acute renal failure, myocardial infarction, and deep vein thrombosis, among patients aged 70 and above undergoing RYGB but not SG. Observed differences may suggest that SG is the preferred procedure for elderly patients with organ‐specific risk factors. These findings were not supported by our meta‐analysis because the surgical techniques examined did not differ significantly in terms of their complications.

Similarly, Smith et al. conducted a study on 641 septuagenarians aged over 70 who underwent non‐revisional bariatric surgery, including 439 (68.5%) SG and 202 (31.5%) RYGB.^22^ Regarding RYGB, septuagenarians exhibited a notably higher incidence of any complication (14.6% vs. 10.7%; *p* < 0.05) in comparison to individuals aged 45–69 years. In the case of SG patients, those aged over 70 years manifested an elevated occurrence of any complication (7.4% vs. 5.2%; *p* < 0.05) and severe complications (2.7% vs. 1.5%; *p* < 0.05). There existed no statistically significant variance in severe complications or mortality between patients aged over 70 years undergoing RYGB or SG and those aged 45–69 years. The findings of Pechman et al. and Smith et al. are in line with smaller studies and our results. An important exception is the mortality rate, which in our review is equal to 1%, significantly higher than in younger patients treated with bariatric surgeries.

When assessing the effectiveness of bariatric surgery in people over 70 years of age, the barrier to a full assessment of the results was the variety of factors assessing the effectiveness of treatment and the different time intervals for measuring them. Comparing the coefficients of change in BMI, %EWL, and percentage of total weight loss (%TWL), we discovered that the most frequently repeated coefficient was %EWL measured 1 year after surgery (10 studies) and %EWL measured half a year after surgery (6 studies). In the case of the assessment of obesity complications, all studies reported how many patients were preoperatively diagnosed with T2D and HT, but only in nine studies was it possible to extract the remission rate. When evaluating postoperative complications across studies, we tried to gather them into larger groups whenever possible to present the problem of postoperative complications more clearly.

After assessing the results of our review, we believe that age should not be considered an absolute contraindication for bariatric surgery. Other common surgeries for the elderly have proven to be beneficial for both patients and the healthcare system.^32^, ^33^, ^34^ We believe that if these surgical interventions are deemed clinically indicated, the potential benefits of bariatric surgery should also be considered. Bariatric surgeries for the elderly over 70 years of age can manage concurrent obesity‐related diseases, reduce drug dependency, and potentially enhance overall daily quality of life. Finally, it is crucial to acknowledge that surgical procedures in elderly patients involve heightened risks and that outcomes may not align with the expectations typically observed in younger patient cohorts.

### 4.1 Limitations

Despite the high quality of the publications included in the review, the main limitation was the design of the studies included. All were retrospective in nature, and only a few studies included a placebo group, which significantly reduced their certainty. This, combined with the heterogeneity of the measurements used or the insufficient amount of data, made it impossible to conduct this review in the classic form of meta‐analysis. Moreover, due to high heterogeneity, the synthesized average values, ranges, etc. may not reflect the actual situation that could be determined if the research methodology used was consistent. In several cases, the small population was a limitation. Therefore, the results obtained may not correspond to the results if the cohort was larger. The review also included only studies published in English, excluding potentially relevant articles published in other languages.

## 5 CONCLUSIONS

In conclusion, this systematic review suggests that laparoscopic bariatric surgery is an effective and safe treatment option for septuagenarians. In 70‐year‐olds, RYGB proved to be beneficial compared with SG in weight loss, but none of the analyzed surgical methods stood out in terms of complications. Patients up to 70 years of age are characterized by a slightly better metabolic effect, expressed in improvement in obesity‐related diseases, with significantly lower postoperative morbidity and mortality. Therefore, careful preoperative selection of patients aged >70 years is necessary to ensure optimal treatment outcomes. There is still a lack of comparative studies on bariatric surgery in older people aged >70 years. In the future, further research will be necessary to clarify the place of bariatric surgery in the treatment of 70‐year‐olds and to assess its long‐term impact on quality of life.

## CONFLICT OF INTEREST STATEMENT

No conflict of interest was declared.
